# Brain-kidney crosstalk in subarachnoid hemorrhage patients

**DOI:** 10.62838/jccm-2026-0037

**Published:** 2026-07-27

**Authors:** Elisabete Monteiro, Sofia Rocha e Silva, Silvina Barbosa, Isaac Barroso, Cláudia Camila Dias, Marek Czosnyka, José Artur Paiva, Celeste Dias

**Affiliations:** Department of Intensive Care Medicine, Unidade Local de Saúde São João, Porto, Portugal; Faculdade de Medicina da Universidade do Porto (FMUP), Porto, Portugal; RISE-Health Unit, Faculdade de Medicina da Universidade do Porto (FMUP), Porto, Portugal

**Keywords:** creatinine clearance, autoregulation, pressure reactivity index, augmented renal clearance

## Abstract

**Background:**

A “crosstalk” between brain and kidney after Subarachnoid Hemorrhage (SAH) is suggested. A link can be the vascular autoregulation. It can be evaluated with cerebrovascular Pressure Reactivity index (PRx). It is not clear if augmented renal clearance (ARC) can be used as a marker of kidney dysregulation or if ARC is associated with brain autoregulation. Our aim was to evaluate correlation between ARC and PRx in SAH.

**Methods:**

Pilot study of SAH patients admitted to a Neurocritical Care Unit (NCCU), at tertiary Portuguese hospital, submitted to multimodal brain monitoring. Fifteen patients were included over a period of two years. Daily observations from 15 patients were analysed, during the first 14 days after admission. Creatinine clearance was measured (MCC) using plasma creatinine and urinary creatinine in a 6-hour urine collection. Multimodal brain monitoring data included intracranial pressure, cerebral perfusion pressure, optimal perfusion pressure (optCPP; CPP level which makes autoregulation working optimally) and PRx.

**Results:**

Fifteen patients were included. Median age was 58 and initial Glasgow Coma Score 13, Hunt and Hess classification was ≥3 in 66%, and Fisher was 4 in 100%. ARC is highly prevalent since the admission. There was a positive correlation between the number of days in ICU and CPP (R2=0.37), optimal CPP (R2=0.23), and PRx (R2=0.25), indicating loss of autoregulation over time. There was no significant association between ARC and preserved autoregulation (R2=0.05) suggesting that ARC is itself a manifestation of kidney dysregulation. ARC doesn't correlate with noradrenaline or fluid balance. In multivariate analysis only age has an impact on MCC, with MCC expected to decrease by 1.328 for one year of life.

**Conclusions:**

ARC is highly prevalent in SAH patients during the first two weeks after NCCU admission. Presence of ARC, evaluated with MCC, was not significantly associated with the presence of cerebral autoregulation.

## Introduction

Cerebral autoregulation describes the intrinsic ability of cerebral vessels to maintain constant blood flow despite variations in cerebral perfusion pressure (CPP). One of most important mechanisms governing cerebral blood flow is cerebrovascular pressure reactivity described as the ability of vascular smooth muscle cells to react to changes in transmural pressure [1].

One way to evaluate autoregulation, continuously and at bedside, in patients with acute brain injury, is by using Pressure Reactivity index (PRx) that uses moving correlation coefficients from mean intracranial pressure (ICP) and mean arterial pressure [2]. Higher values of PRx indicates worse autoregulation, and lower (negative value) indicates autoregulation is functioning well. Optimal Cerebral Perfusion Pressure (CPPopt) was defined by Steiner [3] as the value of Cerebral Perfusion Pressure (CPP) corresponding to the lowest PRx value observed, indicating autoregulation working optimally.

After Subarachnoid Hemorrhage (SAH) [4,5] failure of cerebral autoregulation is common and the severity of impaired autoregulation is linked to the severity of the initial primary lesion and secondary hemodynamic disturbances like cerebral vasospasm [6,7,8,9]. Impaired autoregulation has been associated with poor outcome in numerous studies [10,11,12,13].

Brain and kidney share a mechanism of autoregulation that maintains constant blood flow over a wide range of blood pressures [14,15]. However, it is unclear if changes in the autoregulation mechanism of one organ may affect the mechanism of autoregulation in the other organ [16]. Some authors suggested that loss of cerebral autoregulation may coincide with impairment of renal autoregulation [17,18]. After SAH, brain vessels are prone to vasospasm, while renal circulation is free from this phenomenon.

Glomerular filtration rate (GFR) is the best approximation of renal function [19,20] and its estimation is of clinical importance regarding detection of acute kidney injury or augmented renal clearance (ARC). The gold standard measurement of GFR involves inulin clearance by the kidneys but it is complex [20] and, therefore, in clinical practice it is replaced by endogenous markers [21]. Creatinine is the most used [22], by means of its clearance (CrCl) or by estimated formulas [23,24,25,26].

Measured creatinine clearance (MCC) is more accurate [27] than estimated using formulas. It can be assessed using plasma and urine creatinine concentration and urine output. In patients admitted to intensive care units (ICU), shorter time evaluation (for instance 6 hours, as used in this study) is representative of the 24-hours period [28,29]. Augmented renal clearance is defined as CrCl >130 ml/min/1.73 m^2^ [30]. It has been demonstrated in Traumatic Brain Injury (TBI) [31,32] and spontaneous Subarachnoid Hemorrhage (SAH) [33,34] and in patients admitted to Intensive Care Units with acute brain injury [27].

In both diseases (TBI and SAH) the use of aggressive fluid resuscitation and vasoactive drugs [35,36] can contribute to ARC.

In a retrospective study of 18 patients with traumatic brain injury [37], Dias et al, suggested that better cerebrovascular autoregulation was significantly associated with renal hyperfiltration and potentially better outcomes. It should be noted that the data in the literature on ARC and PRx come mainly from TBI, not SAH, and the existing studies used estimates and not the measured creatinine clearance. Patients' gains regarding a clear determination of ARC include better drugs adjustments namely antibiotics and anti-epileptics. Establishing a relationship between brain autoregulation and ARC improves the understanding the pathophysiology of a disease with high socioeconomic burden.

In this study, our objective was to document positive association between ARC (measured not estimated) and intact autoregulation evaluated with PRx, in patients with non-traumatic SAH, submitted to brain multimodal monitoring. A secondary aim is to evaluate the contribution of noradrenaline dose and fluid balance to ARC in these patients.

## Materials and Methods

### Patient selection

In this observational single-center, pilot study, we prospectively included patients with severe acute spontaneous aneurysmal SAH requiring multimodal brain monitoring, admitted to the Neurocritical Intensive Care Unit (ICU) of Centro Hospitalar e Universitário São João (tertiary level, university-affiliated hospital, Porto, Portugal), recruited from March 2014 until December 2016. Clinical and computer data were then analyzed. Patients less than 18 years old, pregnant females, those with pre-existing renal failure or acute renal failure and with expected survival less than three days were excluded.

All eligible patients were taking into account during the recruitment period, but we excluded those with missing data concerning neuromonitoring and creatinine measurement from the analysis. Patients requiring multimodal monitoring were managed according to local protocols applicable to all patients with severe SAH.

Patients' ICU follow was up to 14 days. The Institutional Research Ethics Committee approved the study (CES 306.13), and a written consent was obtained from a legal representative. All patients were included only after obtaining consent.

### Data collection and analysis

Demographic and clinical variables were recorded such as age, gender, Glasgow Coma Scale at first aid and at hospital admission. Disease severity and mortality prediction on admission were calculated using SAPS II score (Simplified Acute Physiology Score) [38], and clinical and radiological severity scales Hunt and Hess [39] and Fisher [40] were also calculated. Regarding systemic monitoring, all patients had a Philips Intellivue® multiparameter monitor that allowed bedside continuous acquisition of ECG, heart rate, respiratory rate, invasive arterial blood pressure (ABP), pulse oximetry and end-tidal CO2. Regarding multimodal brain monitoring [41,42] all patients were managed with Intracranial pressure (ICP), Cerebral Perfusion Pressure (CPP), pressure Reactivity Index (PRx), optimal CPP (optCPP) achieved with PRx (optCPPPRx) and the difference between CPP given to patient and optCPPPRx (CPP-optCPPPRx). PRx was used for continuous evaluation of autoregulation [43] at bedside, individualizing therapy. Calculation of optCPP and ccontinuous data recording were achieved with ICM+®software, (http://www.neurosurg.cam.ac.uk/icmplus). According to Neurocritical Care ICU protocol, optCPP is a target value for CPP and it is achieved with fluids and vasoactive drugs. Variables of brain multimodal monitoring between 1 am and 7 am were recorded and analyzed. Daily blood tests were performed (at 7 am), for chemistry assessments, namely serum creatinine. All patients had an indwelling urinary catheter, and a 6-hour urine sample was collected between 1 am to 7 am to measure urine creatinine, during the first 14 days after admission. Urine output was recorded. Urine and serum creatinine levels were assessed using an Olympus AU5400 Clinical Chemistry analyzer from Beckman Coulter.

Augmented renal clearance was defined as a CrCl above 130 mL/min/1.73 m^2^ [30]. Creatinine clearance can be calculated by measuring plasmatic creatinine and urinary creatinine (MCC) or by formulas that can estimate CrCl such as Cockcroft–Gault (CG), abbreviated Modification of Diet in Renal Disease Study Equation (MDRD) and Chronic Kidney Disease Epidemiology Collaboration (CKD-EPI) [27].

### Statistical methods

Continuous variables were expressed as mean values with standard deviation (mean ± SD) or medians and interquartile range (median; (IQR)). Categorical variables were presented as a count (n) or percentage (%).

When testing a hypothesis about continuous variables, nonparametric tests Mann-Whitney or Kruskall-Wallis were used as appropriate, considering normality assumptions and the number of groups compared. When testing a hypothesis about categorical variables a chi-square test and Fisher's exact test were used, as appropriate.

Generalized linear mixed models (GLMMs) were therefore developed to quantify the impact of MCC in each patient. The fixed-effect factor covariates in our model were age but it was adjusted to gender, number of days, CPP, PRx, optimal CPP by using PRx (optCPPPRx) and (CPP-optCPPPRx) because these factors were considered clinically relevant. The random variable was the patient.

The significance level used was 0.05. Statistical analysis was performed using the software Statistical Package for the Social Sciences v. 24 and R and R Studio (R version 4.2.1., The R Foundation for Statistical Computing, Vienna, Austria).

## Results

### Demographic data

**Fig. 1.**
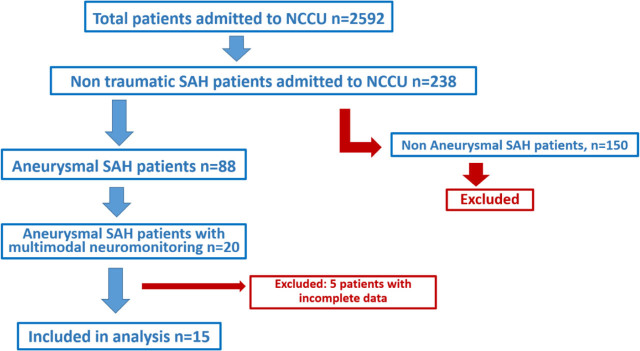
Flowchart of study population Legend: SAH – Subarachnoid Hemorrhage; TBI– Traumatic Brain Injury.

Among the 2592 patients admitted at NCCU, 238 patients had the diagnosis of SAH. An aneurysm was identified in 88 but only 20 had complete multimodal neuromonitoring and informed consent. A flowchart of the population studied is presented in Figure 1. Fifteen patients were enrolled, 5 males (33%) and 10 female (67%). Median age was 58 (IQR 37-62). Median SAPS II was 47 (Median SAPS II mortality 47%) and Glasgow Coma Scale at hospital (GCS) was 12. Median ICU length of stay (LOS) and hospital LOS were 23 (IQR 15-29) and 40 (IQR 29-64) days, respectively. Characteristics of patients are reported in Table 1.

**Table 1.** Demographic and Baseline Patients' Characteristics (n=15)

Gender, male n (%)	5 (33)
Age, median (P25-P75)	58 (37–62)
LOS in ICU, median (P25-P75)	23 (15–29)
LOS in hospital, median (P25-P75)	40 (29–64)
SAPS II, median (P25-P75)	47 (28–56)
SAPS II mortality %, median (P25-P75)	39 (9–60)
GCS at first aid, median (P25-P75)	13 (8–15)
GCS at hospital, median (P25-P75)	12 (3–14)
SAH H&H, n (%)	
I	0 (0)
II	5 (33)
III	3 (20)
IV	2 (13)
V	5 (33)
SAH Fisher, n (%)	
1	0 (0)
2	0 (0)
3	0 (0)
4	15 (100)
SAH vasospasm, n (%)	8 (53)
Mortality at ICU discharge, n (%)	2 (13)
Creatinine Clearance at admission (ml/min/1.73 m^2^), median (P25-P75)	
MCC	139 (120–243)
CG	104 (75–155)
MDRD	127 (89–173)
CKD-EPI	112 (83–131)
Vasopressors on day 1 (Noradrenaline) Dose (mcg/kg/min), median (P25-P75)	0.19 (0.07–0.32)
Number of patients on diuretics on day 1, n (%)	2 (15)

GCS – Glasgow Coma Scale; LOS – length of stay; MCC–Measured Creatinine Clearance; SAH H&H – Hunt and Hess classification for Spontaneous Subarachnoid hemorrhage (SAH); SAH Fisher – Fisher classification for SAH; SAPS II – Simplified Acute Physiology Score; MDRD – abbreviated Modification of Diet in Renal Disease Study Equation; CKD-EPI – Chronic Kidney Disease Epidemiology Collaboration

### Creatinine clearance and multimodal brain monitoring data

At ICU admission, MCC (median and P25-P75) was 139 (120–243) ml/min/1.73 m^2^ and this value is higher than those obtained with formulas that estimated CrCl. As seen in Table 1 CrCl evaluated with CG is 104 (75–155), with MDRD is 127 (89–173) and with CKD-EPI is 112 (83–131) ml/min/1.73 m^2^. Differences are maintained between different methods of Cr Cl evaluation over time. It is important to underline that the evaluation of MCC and ARC didn't take in account different syndromes such as diabetes insipidus, inappropriate secretion of antidiuretic hormone or cerebral salt waste syndrome.

**Fig. 2.**
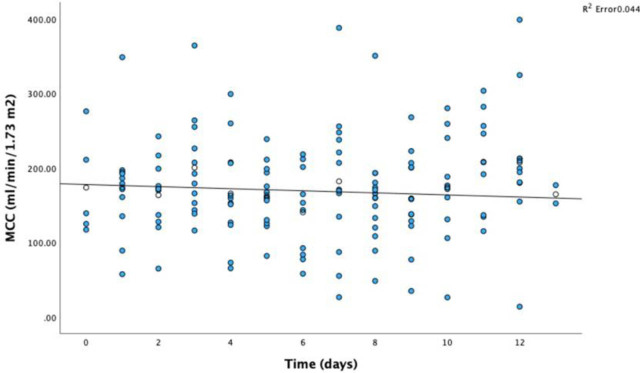
Global Median MCC evolution during the first 14 days of ICU stay Legend: MCC – Measured Creatinine Clearance (mL/min/1.73 m2); Time – length of stay in ICU (days).

Regarding individual evolution of MCC we can observe high values of MCC that are kept high as days go by. There are not well-established different patterns of evolution. Global evolution of the median MCC during the first 14 days of ICU stay can be seen in Figure 2. Values above 130 ml/min/1.73 m^2^ are considered increased. Solid line is a regression linear model indicating very weak dependence of MCC on time after SAH.

Regarding noradrenaline and fluid balance we were not able to demonstrate any association between those variables and ARC. Measured creatinine clearance values are high starting at admission and remain elevated throughout the 14 days. Noradrenaline dose decreases from dose at admission of 0.19 (0.07–0.32) mcg/kg/min to zero at the 14th day. Fluid balance also decreases from +1295 ml at admission to +271 at the last day of follow up.

Concerning multimodal brain monitoring variables evolution over time, CPP, OptCPP and PRx, a regression model was applied, and a positive correlation can be seen between the number of days and CPP (R^2^=0.37), optimal CPP (R^2^=0.23), and PRx (R^2^=0.25). If measurements on consecutive days of these variables are treated as independent, p would be respectively < 0.0001, <0.008 and 0.005.

Pressure reactivity index increasing as days go by, indicates gradual losing autoregulation over time.

**Fig. 3.**
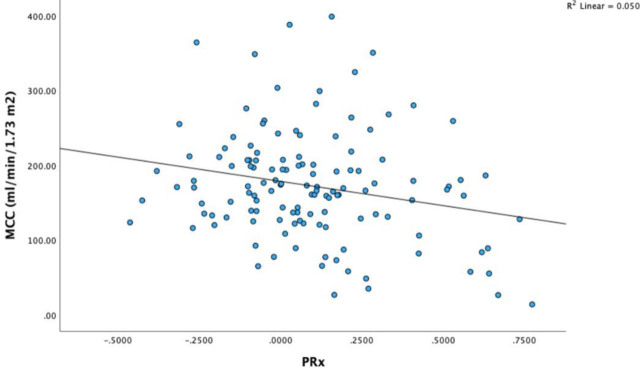
Correlation between MCC and PRx Legend: MCC – Measured Creatinine Clearance (mL/min/1.73 m2); PRx – Pressure reactivity index

In Figure 3. we can observe the absence of correlation between Measured Creatinine Clearance and autoregulation evaluated with PRx. Solid line is a regression linear model indicating very weak dependence between MCC and PRx on time after SAH.

To quantify the impact of different variables on MCC, for each patient, for better accuracy, generalized linear mixed models (GLMMs) were developed.

In univariate analysis, we tested the impact of gender, age, number of days, CPP, PRx, optCPPPRx and (CPP-optCPPPRx) in MCC, therefore on ARC. Only age has an impact on MCC, with MCC decreasing in 1.328 points for an increase in each year age.

In multivariate, mixed models' analysis included obviously age but also other variables considered clinically relevant such as gender, number of days, CPP, PRx, optCPPPRx, (CPP-optCPPPRx), noradrenaline and fluid balance.

No association was found between ARC (evaluated with MCC) and autoregulation (with PRx), and this was kept in a multivariate analysis. GLMMs results are in Table 2.

**Table 2.** Factors associated with creatinine clearance

**Fixed-effect factor**			
	**Coefficient**	**CI 95%**	**p-value**
Gender – Female	15.18	−43.79;74.07	0.615
Age	−1.33	−2.81;0.15	0.080
Number of days (Time)	0.51	−1.61;2.64	0.635
CPP	0.64	−0.30;1.59	0.185
PRx	−18.39	−61.48;23.64	0.393
CPP-optCPPPRx	−0.71	−1.74;0.30	0.169
Noradrenaline	−37.15	−90.77;16.95	0.175
Fluid balance	−0.002	−0.009;0.000	0.632

MCC – Measured Creatinine Clearance; CI 95% – 95% Confidence intervals; CPP – Cerebral Perfusion Pressure; PRx – Pressure Reactivity index; optCPPPRx – optimal CPP evaluated with PRx; CPP-optCPPPRx – CPP given to the patient minus optimal CPP evaluated with PRx.

## Discussions

The first point to underline is the high value of MCC and the high prevalence of ARC in SAH. In fact, these neurocritical severely ill patients present, upon admission, a very high CrCl and that is kept during the following 15 days. MCC is higher than the value estimated with formulas. These findings are consistent with literature despite the paucity of information in patients with SAH [33,34]. In other settings this has been better studied, and more information is available. ARC was studied by Udy [32] in 20 patients with TBI and vasopressors and hypertonic saline were associated with the occurrence of ARC. Later, in 2014, Udy and Baptista [44] evaluated different subgroups of patients (included a subgroup of patients admitted at ICU) and found that 65.1% of them had at least once ARC determination over the 7 days period of monitoring.

The second point to highlight is that no significant correlation was found between ARC and cerebral autoregulation evaluated with PRx. Autoregulation of blood flow describes the ability of a vascular bed to maintain its perfusion constant despite variations in arterial perfusion pressure [5]. This mechanism is present in the brain and kidney and rather absent in other organs. In fact, brain and kidney present a common and unique way to react to fluctuations in blood pressure due to similar small resistance vessels [18] and this may explain the microvascular damage suggested by Thomson and Hakim [45] and called, at that time, arteriolar systemic dysfunction.

In patients with acute brain injury, pathological neurohumoral changes can directly affect kidney autoregulation by increasing sympathetic nervous system activity and by changes in sodium and water balance due to abnormal vasopressin release [26]. Patients with acute brain injury are at risk to acute kidney injury (AKI) due to several easily understood factors, such as hypovolemia, reduced intrarenal glomerular perfusion and exposure to radiocontrast and nephrotoxic drugs. ARC can be justified, after SAH, with several potential mechanisms, including endogenous responses to increased metabolism and solute production, alterations in neurohormonal balance and therapeutic interventions (such as use of vasopressors and fluid resuscitation) [18].

Regarding similitude between cerebral and kidney circulation, in both organs there are regulatory vessels of small resistance. It would be intuitively easy to accept that brain and kidney react in a similar way. A link between kidney and brain in traumatic brain injury patients was reported previously, documented a strong correlation between PRx and estimated creatinine clearance [37].

In the present paper we do not corroborate the previous results for TBI patients, and we documented an absence of correlation between preserved autoregulation and ARC, evaluated by MCC, in patients with SAH. A significant difference between these studies is that in the present study we use MCC and not formulas to estimated CrCl as authors did in TBI patients. Another point to highlight is that SAH, differently from TBI, is a purely vascular disease and vasospasm observed more frequently after SAH than after TBI, may be a confounding factor. Obviously, vasospasm doesn't happen in renal circulation.

The third relevant point refers to contributing factors to achieve cerebral autoregulation and ARC. We reviewed the contribution of the use of noradrenaline (NA), the fluid balance and the optimal perfusion pressure given to these patients with SAH. In fact, our NCCU protocol advocates the use of fluid and NA [43] to achieve optimal perfusion pressure [43]. However, the missing link contributing to ARC doesn't seem to be the use of NA nor the positive fluid balance since despite decreases in NA and negative fluid balances, ARC remains high throughout the first 14 days of hospitalization. While these findings are divergent from the literature, it's worth noting that those studies do not specifically address a particular subtype of patients with SAH. [35,36,46].

The fourth main issue of this paper is the similar time trend between optimal CPP and ARC after ictus, strengthening the crosstalk between brain and kidney, but were unable to demonstrate a clear association (see Table 2).

PRx increasing over time is also an important issue, stating loss of autoregulation. In fact, in SAH patients, hyperfiltration leads to polyuria, that in turn leads to plasmatic volume depletion and vasospasm [47] may occur and contribute to impaired autoregulation.

Dysfunction of microcirculation can be the ultimate link, and new emerging surrogates (such as the hematocrit-to-hemoglobin ratio) can improve the understanding of brain-kidney crosstalks [48].

## Limitations

This study has several limitations. First it is a single-center study with a small sample of patients. Second, measurement of CrCl is not the best way to evaluate glomerular filtration. It is possible that a selection bias exists namely multimodal monitoring is not applied to all patients with SAH.

A third limitation is that a sample size calculation based on the primary outcome was not provided and this study can be underpowered due to that. Additionally, the use of multivariable regressions in such a small cohort can introduce a bias.

In the future all patients with SAH should have a direct determination of renal clearance and a close monitoring of ARC in addition to continuous bedside autoregulation assessments.

Further additional prospective research and extension to multicenter studies are required.

## Conclusions

In SAH patients, Augmented Renal Clearance, evaluated with MCC, is a very frequent finding at admission to ICU and throughout the first 14 days. ARC was not associated with state of autoregulation in this specific group of patients after SAH. Noradrenaline dose and fluid balance don't seem responsible for ARC. Autoregulation, evaluated with PRx, is lost over the days.
